# Supplementary material for: circTP63 functions as a ceRNA to promote lung squamous cell carcinoma progression by upregulating FOXM1
Source: Nat Commun. 2019 Jul 19;10:3200. doi: 10.1038/s41467-019-11162-4 (PMC6642174; doi:10.1038/s41467-019-11162-4)
Supplement: Supplementary file 1 — Supplementary Information [file 41467_2019_11162_MOESM1_ESM.pdf]

Supplementary Information

c*ircTP63* functions as a ceRNA to promote lung squamous cell carcinoma progression by upregulating FOXM1

Cheng et al.

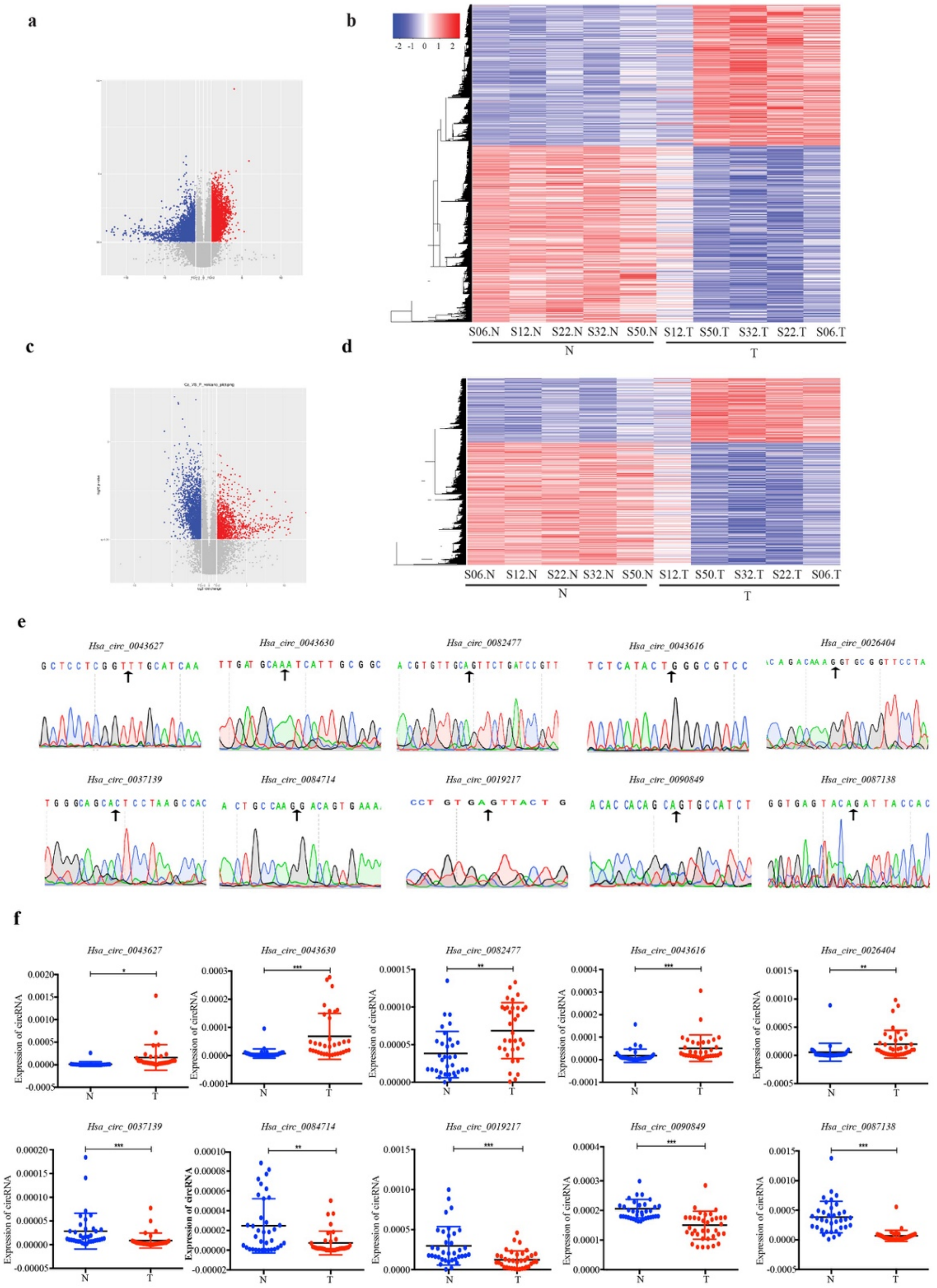

Supplementary Figure 1. Differential expression of circRNAs and mRNAs in LUSC. (**a-b**) A volcano plot and a heatmap show 7081 differentially expressed circRNAs in 5 paired samples of LUSC by SBC Human ceRNA Array analysis. Cutoff criterion was foldchange of ≥2 or ≤-2, *p* value ≤0.05. (**c-d**) A volcano plot and a heatmap show 2832 differentially expressed mRNAs in 5 paired sample of LUSC by SBC Human ceRNA Array analysis. Cutoff criterion was foldchange of ≥2 or ≤-2, *p* value ≤0.05. (**e**) Sanger sequencing identified the back-splice junction of circRNAs. (**f**) qRT-PCR analysis for the expression of circRNAs in 35 LUSC tissuses and corresponding adjacent nontumorous tissues. Upper panel: 5 upregulated circRNAs; Lower panel: 5 downregulated circRNAs. The error bars (**f**) represent s.d (n=35); **p*<0.05; ***p*<0.01; ****p*<0.001, paired t test.

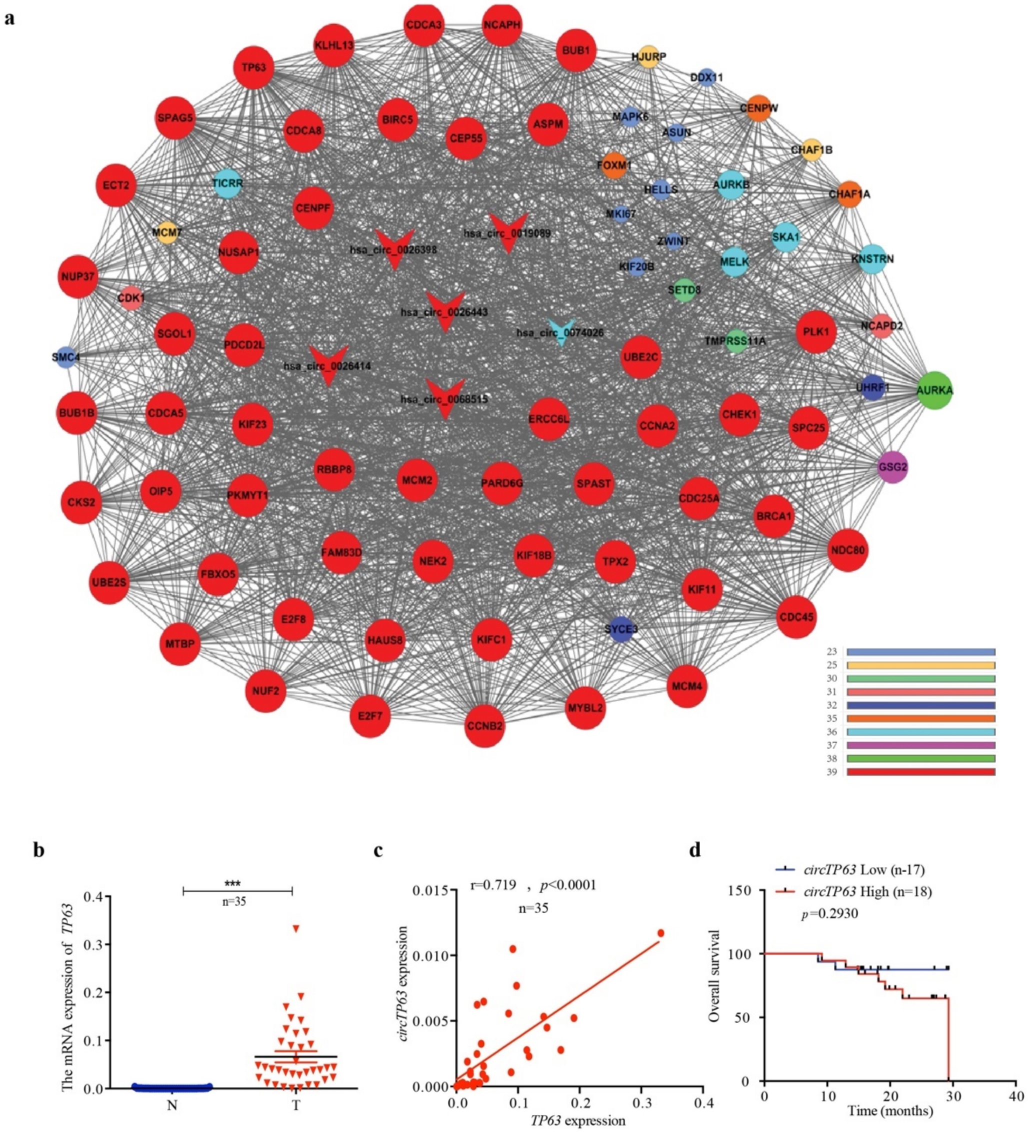

Supplementary Figure 2. The correlation of *circTP63* with cell cycle-related genes, *TP63* expression, and prognosis in LUSC. (**a**) Co-expression network of 6 significant circRNAs with 79 cell cycle-related mRNAs in LUSC. A round node represents a protein-coding gene and the arrow node represents circRNAs. Lines between two nodes indicate interactions between two genes. The color and size of round and arrow nodes represent numbers of interaction genes. (**b**) qRT-PCR analysis for the expression of *TP63* in the 35 LUSC tissues. (**c**) Correlation analysis of *circTP63* and *TP63* mRNA. (**d**) Preliminary Kaplan-Meier analysis for the correlation between *circTP63* expression and overall survival in LUSC patients. The error bars (**b**) represent s.d (n=35); ****p*<0.001, paired t test.

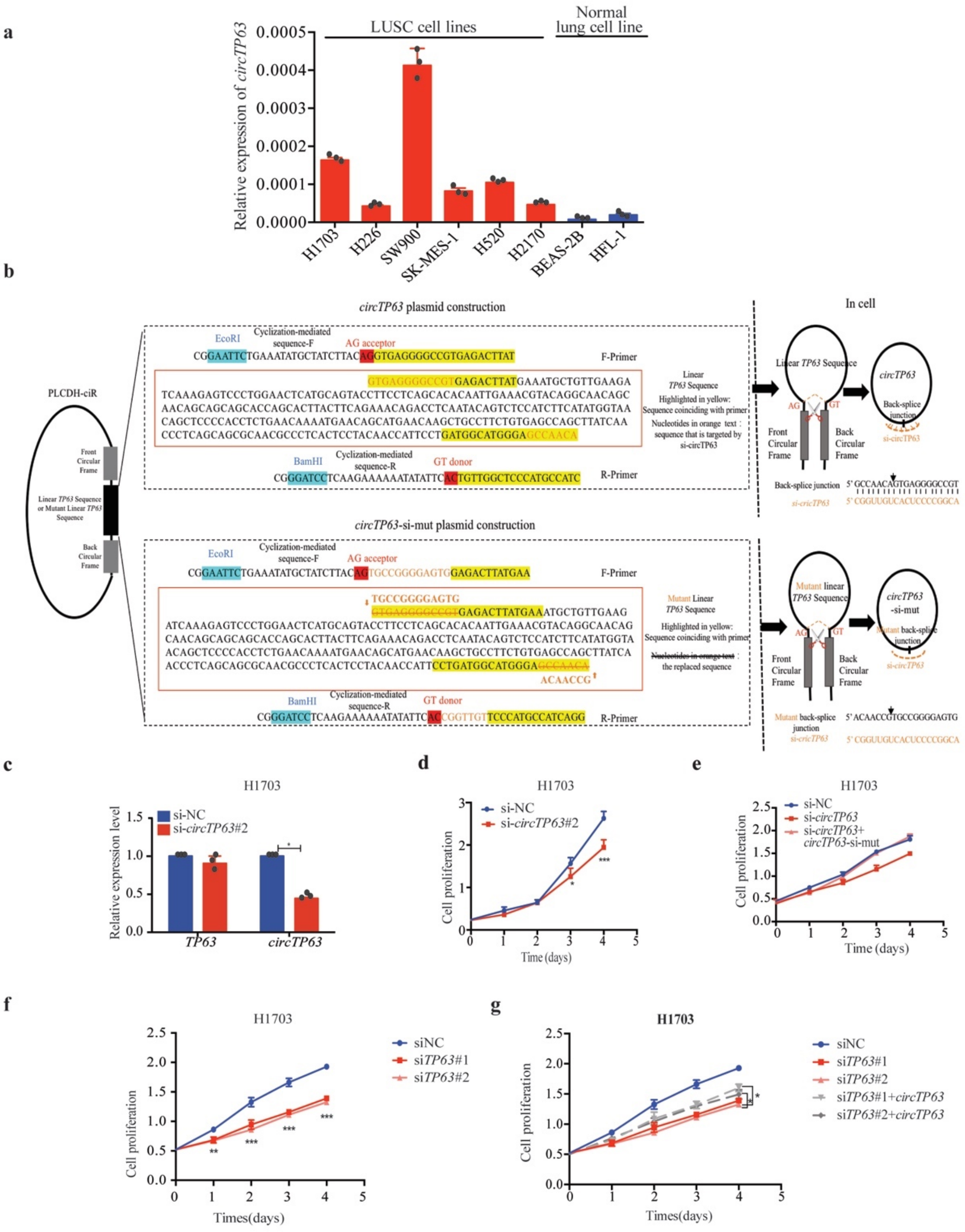

Supplementary Figure 3. Proliferation-promoting effect of *circTP63*. (**a**) Expression of *circTP63* in 6 LUSC cell lines and 2 normal cell lines (a human bronchial epithelial cell line BEAS-2B and a human fetal lung fibroblast cell line HFL-1). (**b**) A sketch map of *circTP63* or *circTP63*-si-mut plasmid construction. (**c**) qRT-PCR analysis for the efficiency of si-*circTP63*#2. (**d**) Proliferation assay for H7103 cells with *circTP63* knockdown by si-*circTP63*#2. (**e**) Proliferation assay for the rescue effect of *circTP63* with a muant back splice junction. (**f**) Proliferation assay for H1703 cells with *TP63* knockdown. (**g**) Proliferation assay for H1703 cells with *TP63* knockdown and *circTP63* overexpression. The error bars (**a**, **c-g**) represent s.d (n=3); **p*<0.05; ***p*<0.01; ****p*<0.001, two-tailed t-test.

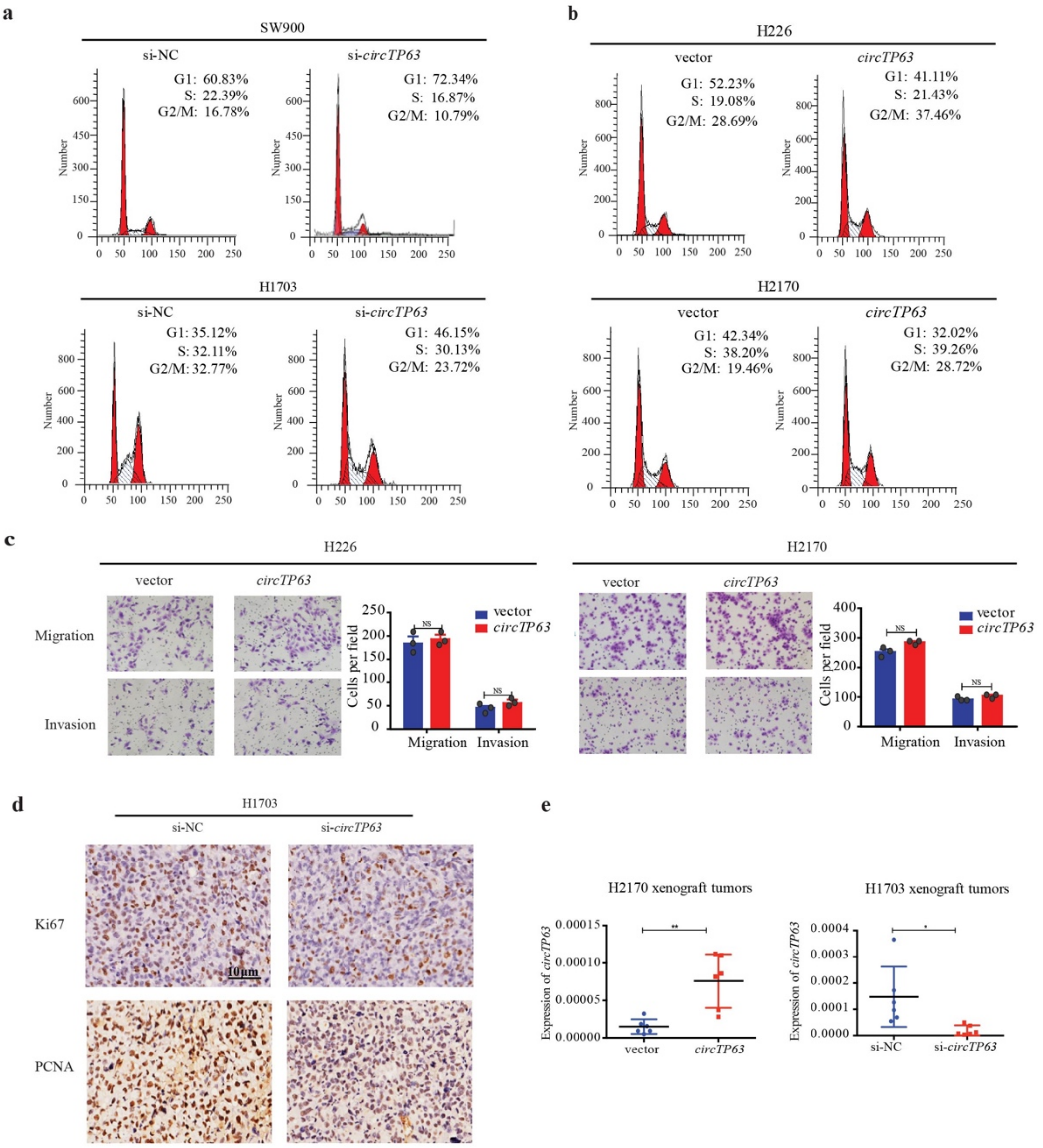

Supplementary Figure 4. ***circTP63*** **promotes cell cycle entry but has no effect on migration and invasion in LUSC cells.** (**a**) Typical pictures showed knockdown of *circTP63* increased the number of cells in the G1 phase, and decreased the number of cells in the G2/M phase. (**b**) Typical pictures showed overexpression of *circTP63* decreased the number of cells in the G1 phase, and increased the number of cells in the G2/M phase. (**c**) Effect of *circTP63* on migration and invasion of LUSC cells. Images were taken by 200× magnification. (**d**) Immunohistochemical staining for cell proliferation markers. (**e**) qRT-PCR analysis for *circTP63* expression in subcutaneous xenograft tumors. The error bars (**c, e**) represent s.d (in **c**, n=3; in **e**, n=6). *p<0.05; **p<0.01; NS means no significant difference, two-tailed t-test.

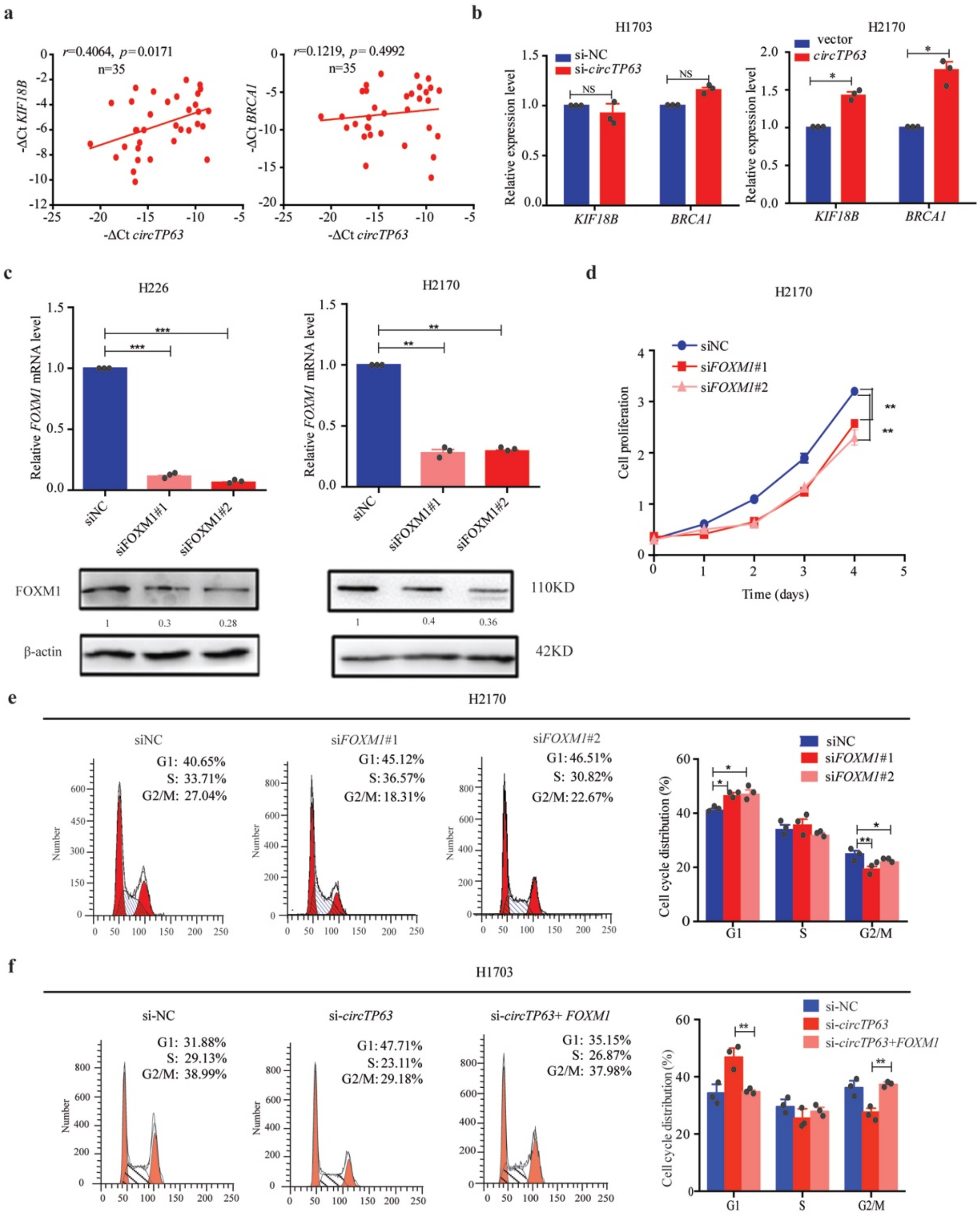

Supplementary Figure 5. *circTP63* promotes cell cycle entry through upregulating FOXM1. (**a**) Analysis for expression correlation between *circTP63* and *KIF18B*, *BRCA1*, respectively, in tumorous tissues of the 35 LUSC patients. (**b**) Detection of mRNA levels of *KIF18B* and *BRCA1* in H1703 cells with *circTP63* knockdown and in H2170 cells with *circTP63* overexpression. (**c**) Efficiency of *FOXM1* knockdown by siRNAs in H226 and H2170 cells. (**d**) Cell proliferation analysis of H2170 cells with silencing *FOXM1*. (**e**) Cell cycle analysis of H2170 cells with silencing *FOXM1*. (**f**) Cell cycle analysis for H1703 cells with *circTP63* knockdown and *FOXM1* overexpression. The error bars (**b-f**) represent s.d (n=3). **p*<0.05; ***p*<0.01; ****p*<0.001; NS means no significant difference, two-tailed t-test. Source data are provided as a Source Data file.

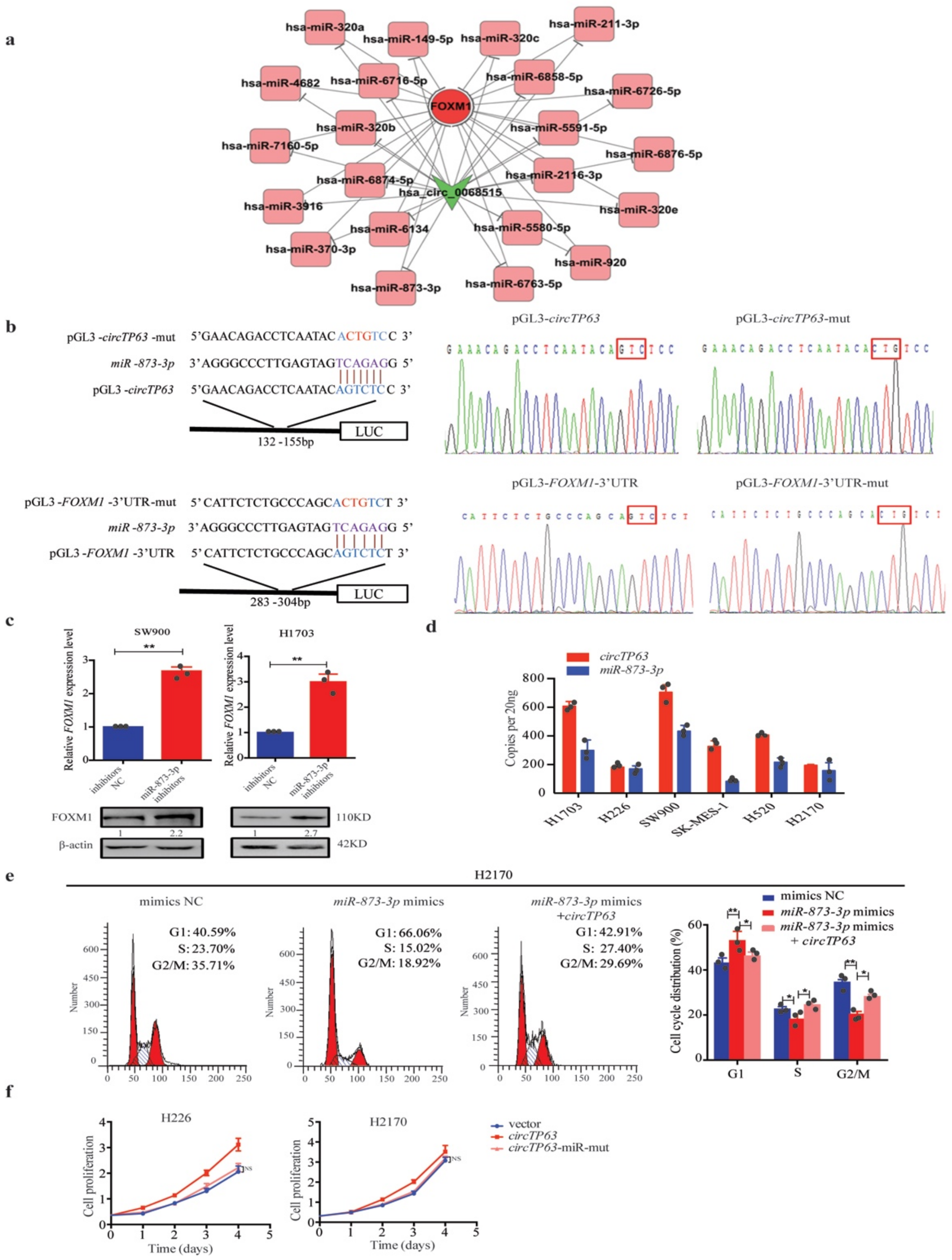

Supplementary Figure 6. *circTP63* serves as a sponge for *miR-873-3p*. (**a**) ceRNA analysis for *circTP63* by cytoscape. The arrow node, circle node and square nodes represent *circTP63* (*hsa_circ_0068515*), *FOXM1* and 22 miRNAs, respectively. Relationship between two nodes is connected with lines. (**b**) Schematic model and Sanger sequencing for wild type or mutant transcripts of *circTP63* or *FOXM*1 3'UTR luciferase reporters. (**c**) Expression of *FOXM1* in SW900 and H1703 cells transfected with *miR-873-3p* inhibitors or NC. (**d**) Abosute expression levels of *circTP63* and *miR-873-3p* in LUSC cell lines. (**e**) Cell cycle analysis for H2170 cells transfected with *miR-873-3p* mimics or co-transfected with *miR-873-3p* mimics and *circTP63*. (**f**) Cell proliferation assay for the effect of *circTP63* with a muant *miR-873-3p* bind site. The error bars (**c-f**) represent s.d (n=3). **p*<0.05; ***p*<0.01; NS means no significant difference, two-tailed t-test. Source data are provided as a Source Data file.

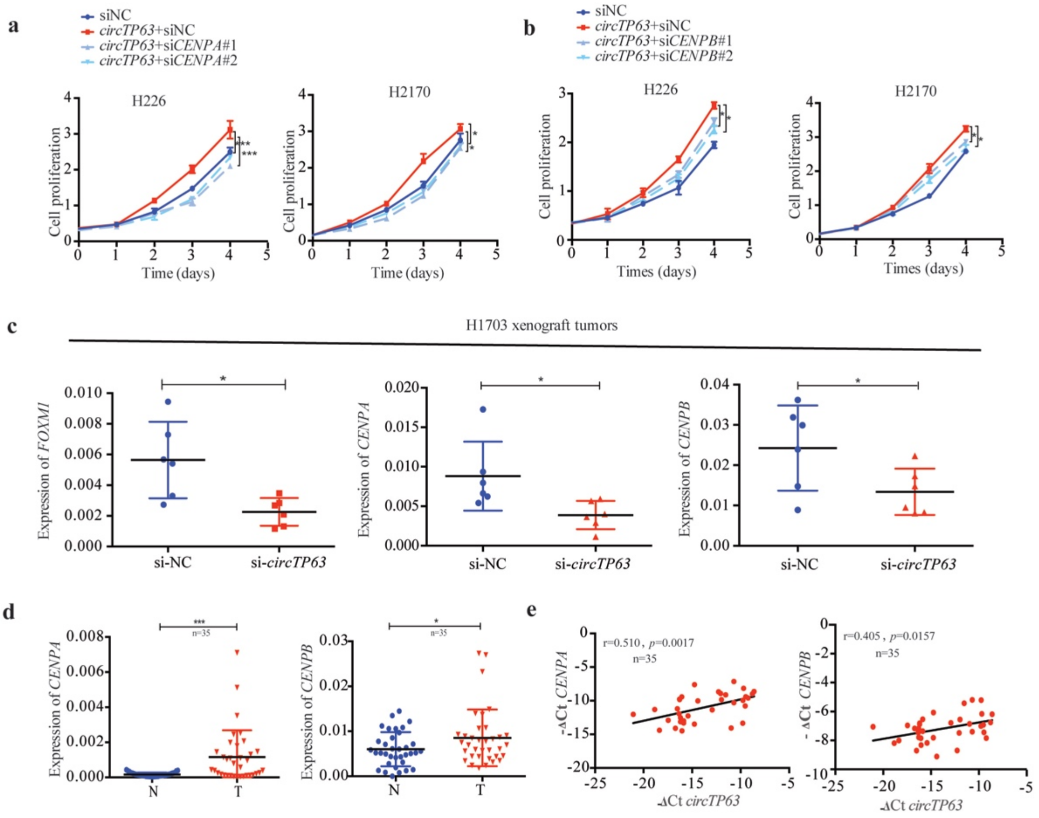

Supplementary Figure 7. Role of FOXM1-CENPA/B pathway in *circTP63*-mediated proliferation of LUSC cells. (**a-b**) Cell proliferation assay for the LUSC cells with *circTP63* overexpression as well as *CENPA* knockdown (**a**) or *CENPB* knockdown (**b**). (**c**) qRT-PCR analysis for *FOXM1*, *CENPA*, and *CENPB* in subcutaneous xenograft tumors of H1703 cells with *circTP63* knockdown. (**d**) Expression levels of *CENPA* and *CENPB* in the 35 paired samples of LUSC. (**e**) Correlation analysis between *circTP63* with *CENPA* or *CEBPB* in the tumorous tissues of the 35 LUSC patients. The error bars (**a-e**) represent s.d (in **a** and **b**, n=3； in **c**, n=6; in **d** and **e**, n=35). *p<0.05; ***p<0.001, two-tailed t-test.

Supplementary Table 1

The top 100 dysregulated circRNAs in 5 paired samples of LUSC

circRNA	Gene symbol	Fold change	*p*-value
Upregulation (n=50)			
hsa_circ_0043610	*KRT14*	2854.766	0.00753
hsa_circ_0026420	*KRT6A*	2141.208	0.009315
hsa_circ_0043609	*KRT14*	1334.51	0.018929
hsa_circ_0043627	*KRT16*	1136.779	0.016823
hsa_circ_0074026	*PITX1*	1106.578	0.001808
hsa_circ_0043630	*KRT16*	987.9269	0.019907
hsa_circ_0074027	*PITX1*	739.6353	0.002523
hsa_circ_0082477	*AKR1B10*	700.8238	0.024095
hsa_circ_0043616	*KRT14*	681.7769	0.02119
hsa_circ_0026443	*KRT5*	643.4123	0.00921
hsa_circ_0026407	*KRT6B*	512.5006	0.016649
hsa_circ_0026404	*KRT6B*	483.758	0.013964
hsa_circ_0043632	*KRT17*	462.0064	0.005137
hsa_circ_0043622	*KRT14*	350.5734	0.017167
hsa_circ_0043614	*KRT14*	334.4521	0.031321
hsa_circ_0026398	*KRT6B*	326.4623	0.008226
hsa_circ_0026414	*KRT6C*	319.3862	0.006636
hsa_circ_0043637	*KRT17*	278.824	0.003269
hsa_circ_0026400	*KRT6B*	257.9169	0.013987
hsa_circ_0043634	*KRT17*	249.7343	0.002863
hsa_circ_0043635	*KRT17*	245.9821	0.003138
hsa_circ_0026437	*KRT5*	242.0897	0.013784
hsa_circ_0026421	*KRT6A*	218.6362	0.016783
hsa_circ_0014235	*S100A2*	210.0535	0.008971
hsa_circ_0043638	*KRT17*	200.1379	0.006838
hsa_circ_0043621	*KRT14*	197.8297	0.02071
hsa_circ_0026452	*KRT5*	183.6982	0.012624
hsa_circ_0026454	*KRT5*	181.5856	0.015912
hsa_circ_0043629	*KRT16*	176.4444	0.02188
hsa_circ_0026403	*KRT6B*	169.934	0.016725
hsa_circ_0026441	*KRT5*	167.1475	0.015346
hsa_circ_0043613	*KRT14*	161.0897	0.01813
hsa_circ_0068515	*TP63*	153.378	0.014067
hsa_circ_0026428	*KRT6A*	150.1994	0.018911
hsa_circ_0026393	*KRT6B*	148.6008	0.016721
hsa_circ_0026431	*KRT6A*	144.5374	0.016017
hsa_circ_0026429	*KRT6A*	144.0495	0.017653

hsa_circ_0026423	*KRT6A*	136.7296	0.013941
hsa_circ_0026425	*KRT6A*	125.5921	0.017885
hsa_circ_0043585	*KRT15*	118.7734	0.017103
hsa_circ_0043644	*KRT17*	114.7275	0.009082
hsa_circ_0026395	*KRT6B*	111.6404	0.014672
hsa_circ_0026446	*KRT5*	105.2151	0.019716
hsa_circ_0026444	*KRT5*	101.0488	0.019315
hsa_circ_0043589	*KRT15*	99.79036	0.019466
hsa_circ_0026460	*KRT5*	96.59003	0.018262
hsa_circ_0025580	*SLCO1B3*	94.62497	0.019939
hsa_circ_0043643	*KRT17*	94.53849	0.017553
hsa_circ_0043626	*KRT16*	86.0253	0.017768
hsa_circ_0043584	*KRT15*	82.41203	0.025537
Downregulation (n=50)			
hsa_circ_0072304	*LIFR*	-9.53543	0.000637
hsa_circ_0005754	*FMN1*	-9.54435	0.001433
hsa_circ_0039727	*CDH5*	-9.55731	0.000401
hsa_circ_0013226	*ARHGAP29*	-9.59039	0.001095
hsa_circ_0026353	*NR4A1*	-9.60707	0.017325
hsa_circ_0017839	*ITGA8*	-9.63289	0.005525
hsa_circ_0056946	*NOSTRIN*	-9.83101	0.002521
hsa_circ_0083381	*DLC1*	-9.83574	0.000893
hsa_circ_0075045	*DUSP1*	-10.135	0.000358
hsa_circ_0074574	*GPX3*	-10.1455	0.001908
hsa_circ_0083383	*DLC1*	-10.1493	0.000308
hsa_circ_0056510	*NCKAP5*	-10.4052	1.06E-05
hsa_circ_0069996	*AREG*	-10.4186	0.008294
hsa_circ_0084710	*PREX2*	-10.4374	0.001236
hsa_circ_0045513	*ABCA6*	-10.4528	0.003574
hsa_circ_0032150	*RHOJ*	-10.4868	0.000566
hsa_circ_0056926	*SCN7A*	-10.5103	0.000669
hsa_circ_0087741	*NR4A3*	-10.6079	0.015387
hsa_circ_0026352	*NR4A1*	-10.6561	0.015222
hsa_circ_0075043	*DUSP1*	-10.7475	0.000278
hsa_circ_0072306	*LIFR*	-10.9234	0.001172
hsa_circ_0072300	*LIFR*	-10.9404	0.000547
hsa_circ_0072298	*LIFR*	-10.9543	0.001064
hsa_circ_0069582	*LIMCH1*	-11.0655	0.002271
hsa_circ_0061233	*None*	-11.2123	0.000836
hsa_circ_0039726	*CDH5*	-11.3175	0.0016
hsa_circ_0087744	*NR4A3*	-11.4197	0.031882
hsa_circ_0069997	*AREG*	-11.7235	0.010089

hsa_circ_0074576	*GPX3*	-11.8651	0.001728
hsa_circ_0084709	*PREX2*	-11.9629	0.001121
hsa_circ_0084711	*PREX2*	-12.0472	0.002769
hsa_circ_0019088	*ANKRD1*	-12.174	4.33E-05
hsa_circ_0070442	*MMRN1*	-13.1166	0.005415
hsa_circ_0074575	*GPX3*	-13.3376	0.002636
hsa_circ_0084712	*PREX2*	-13.4277	0.000693
hsa_circ_0019069	*SLC16A12*	-13.4336	5.40E-05
hsa_circ_0087138	*PGM5*	-13.6256	0.002462
hsa_circ_0090849	*ALAS2*	-13.9412	0.014049
hsa_circ_0084713	*PREX2*	-13.9938	0.001452
hsa_circ_0023604	*ARRB1*	-14.1028	0.000562
hsa_circ_0026354	*NR4A1*	-14.3519	0.013816
hsa_circ_0079732	*INMT-FAM188B*	-14.6299	0.000399
hsa_circ_0019217	*RBP4*	-16.5003	0.000622
hsa_circ_0084714	*PREX2*	-17.0602	0.001629
hsa_circ_0037140	*HBA1*	-17.3322	0.029569
hsa_circ_0084715	*PREX2*	-18.0297	0.001342
hsa_circ_0037139	*HBA2*	-18.909	0.006165
hsa_circ_0024888	*ADAMTS8*	-22.0075	0.004402
hsa_circ_0037141	*HBA1*	-23.9802	0.010467
hsa_circ_0019089	*ANKRD1*	-59.004	2.08E-06

Abbreviation：circRNA referred to circRNA name in circBase

Supplementary Table 2

Predicted 25 co-expression mRNAs with *circTP63*

Number	Genes	Pearson value	*p*-value	FDR
1	*FOXM1*	0.993904	0.008187	0.030816
2	*KIF18B*	0.99323	6.68E-04	0.021859
3	*BRCA1*	0.992816	7.30E-04	0.021859
4	*TP63*	0.992346	2.01E-05	0.013445
5	*SPAG5*	0.991909	8.73E-04	0.021859
6	*CEP55*	0.990544	0.001102	0.022235
7	*CDCA5*	0.989784	0.001238	0.022235
8	*NEK2*	0.985239	0.002148	0.023783
9	*ECT2*	0.983306	0.002583	0.024723
10	*KIF11*	0.981586	0.002991	0.025701
11	*PDCD2L*	0.97939	0.003541	0.02669
12	*TPX2*	0.976924	0.004193	0.02781
13	*BIRC5*	0.975767	0.004512	0.028278
14	*SGOL1*	0.973454	0.005171	0.028793
15	*CDC45*	0.973204	0.005244	0.028793
16	*RBBP8*	0.968386	0.006716	0.030095
17	*PKMYT1*	0.967413	0.007027	0.030397
18	*NCAPH*	0.966559	0.007304	0.030397
19	*BUB1*	0.965084	0.007791	0.0306
20	*CHAF1A*	0.964993	0.007821	0.0306
21	*CDCA3*	0.963925	0.00818	0.030816
22	*MCM4*	0.961865	0.008888	0.031409
23	*ASPM*	0.961181	0.009127	0.031607
24	*SPAST*	0.960312	0.009435	0.031724
25	*CENPF*	0.958882	0.009947	0.031741

Supplementary Table 3
Fold changes of the 25 predicted co-expression mRNAs

Number	Probe name	Gene symbol	Gene description	Fold change	*p*-value
1	LNCV6_144206_PI430048170	*TP63*	tumor protein p63	195.4639	0.013497
2	LNCV6_134619_PI430048170	*CDCA3*	cell division cycle associated 3	8.936355	0.003282
3	LNCV6_133200_PI430048170	*FOXM1*	forkhead box M1	8.520035	0.008198
4	LNCV6_140468_PI430048170	*CDC45*	cell division cycle 45	8.280611	0.012066
5	LNCV6_134615_PI430048170	*CDCA5*	cell division cycle associated 5	7.406359	0.004658
6	LNCV6_128776_PI430048170	*ECT2*	epithelial cell transforming 2	7.258899	0.008691
7	LNCV6_135924_PI430048170	*BIRC5*	baculoviral IAP repeat containing 5	7.180612	0.005952
8	LNCV6_129674_PI430048170	*TPX2*	TPX2 microtubule-associated 2	6.48864	0.00616
9	LNCV6_141039_PI430048170	*CEP55*	centrosomal protein 55kDa	6.449065	0.012507
10	LNCV6_129072_PI430048170	*KIF18B*	kinesin family member 18B	6.074072	0.002454
11	LNCV6_97952_PI430048170	*CENPF*	centromere protein F	5.971249	0.006244
12	LNCV6_141614_PI430048170	*NEK2*	NIMA-related kinase 2	5.944839	0.004781
13	LNCV6_139014_PI430048170	*MCM4*	minichromosome maintenance complex component 4	5.928819	0.010275
14	LNCV6_94130_PI430048170	*ASPM*	asp (abnormal spindle) homolog microcephaly associated (Drosophila)	5.306801	0.005909
15	LNCV6_68652_PI430048170	*PKMYT1*	protein kinase membrane associated tyrosine/threonine 1	5.243974	0.01358
16	LNCV6_144638_PI430048170	*KIF11*	kinesin family member 11	4.295099	0.008109
17	LNCV6_135046_PI430048170	*NCAPH*	non-SMC condensin I complex subunit H	3.980606	0.016877

18	LNCV6_135696_PI430048170	*SPAG5*	sperm associated antigen 5	3.914297	0.004789
19	LNCV6_72751_PI430048170	*SGOL1*	shugoshin-like 1	3.786106	0.005129
20	LNCV6_130916_PI430048170	*BUB1*	BUB1 mitotic checkpoint serine/threonine kinase	3.618905	0.02736
21	LNCV6_135596_PI430048170	*BRCA1*	breast cancer 1 early onset	3.31012	0.020769
22	LNCV6_132257_PI430048170	*CHAF1A*	chromatin assembly factor 1 subunit A (p150)	3.088558	0.020621
23	LNCV6_131475_PI430048170	*RBBP8*	retinoblastoma binding protein 8	2.907967	0.027138
24	LNCV6_139787_PI430048170	*SPAST*	spastin transcript variant 1	2.612743	0.020496
25	LNCV6_128612_PI430048170	*PDCD2L*	programmed cell death 2-like	2.145092	0.021823

Supplementary Table 4

Predicted 22 miRNAs binding to *circTP63* and *FOXM1*

Number	miRNA	*circTP63*_ miRNA energy	*circTP63*_ miRNA score	*circTP63*_ miRNA binding sit start	*circTP63*_ miRNA binding site end	miRNA sequence	miRNA_ *FOXM1* energy	miRNA_ *FOXM1* score	miRNA_ *FOXM1* binding site start	miRNA_ *FOXM1* binding site end
1	*hsa-miR-149-5p*	-20.57	145	271	294	ucuggcuccgugucuucacuccc	-18.31	144	894	917
2	*hsa-miR-873-3p*	-20.72	154	132	155	ggagacugaugaguucccggga	-16.19	150	283	304
3	*hsa-miR-2116-3p*	-21.97	161	268	290	ccucccaugccaagaacuccc	-33.53	150	672	695
4	*hsa-miR-320a*	-23.23	151	211	233	aaaagcugggguugagagggcga	-20.76	160	606	627
5	*hsa-miR-320b*	-22.87	151	211	233	aaaagcuggguugagagggcaa	-19.54	153	849	870
6	*hsa-miR-320c*	-19.88	141	213	233	aaaagcuggguugagagggu	-17.93	157	608	627
7	*hsa-miR-320e*	-18.68	155	214	232	aaagcuggguugagaagg	-18.39	144	609	626
8	*hsa-miR-370-3p*	-26.28	142	224	248	gccugcugggguggaaccuggu	-22.43	140	72	93
9	*hsa-miR-3916*	-22.98	145	48	75	aagaggaagaaauggcugguucuca g	-15.01	142	179	204
10	*hsa-miR-4682*	-19.43	141	37	60	ucugaguuccuggagccuggucu	-26.09	152	779	803
11	*hsa-miR-5580-5p*	-17.77	151	209	230	ugcuggcucauuucauaugugu	-26.55	162	898	918
12	*hsa-miR-5591-5p*	-16.97	145	158	179	ugaggugguaggauguaga	-16.8	148	104	122
13	*hsa-miR-6134*	-16.67	152	167	185	ugaggugguaggauguaga	-22.45	156	587	605
14	*hsa-miR-6716-5p*	-20.59	146	258	277	ugggaaugggggguaagggcc	-20.48	140	271	290
15	*hsa-miR-6726-5p*	-17.84	146	159	179	cgggagcuggggucugcaggu	-24.52	155	102	122
16	*hsa-miR-6763-5p*	-15.68	146	163	181	cuggggaguggcuggggag	-32.57	148	590	609
17	*hsa-miR-6858-5p*	-25.81	145	54	75	gugaggaggggcuggcagggac	-20.18	147	95	114
18	*hsa-miR-6874-5p*	-21.15	149	157	179	auggagcuggaaccagaucaggc	-20.53	143	97	122
19	*hsa-miR-6876-5p*	-18.83	145	51	73	caggaaggagacaggcaguuca	-15.01	144	293	314
20	*hsa-miR-7160-5p*	-22.75	151	57	77	ugcugagguccgggcugugcc	-22.14	159	847	867
21	*hsa-miR-211-3p*	-15.78	140	32	52	gcagggacagcaaaggggugc	-34.26	179	194	214
22	*hsa-miR-920*	-22.45	150	160	179	ggggagcuguggaagcagua	-29.56	169	102	122

Supplementary Table 5

Clinicopathological characteristics of 5 LUSC patients for SBC Human ceRNA Array

Case	Age	Gender	Stage	Smoke	Tumor size (cm)	Vascular invasion	Lymphatic invasion	Differentiation
S06	63	M	IIIA	NO	3*2.5*2.3	NO	YES	Poorly
S12	62	M	IA	NO	4*4*4	NO	NO	Well
S22	50	M	IIA	YES	3.5*3*2.5	NO	YES	Moderately
S32	61	M	IIIA	YES	4*3*2.5	NO	YES	Moderately
S50	70	M	IIA	NO	6*4*7	NO	YES	Moderately

Supplementary Table 6

Clinicopathological information of 35 LUSC patients for qRT-PCR varidation

Age (≤60:>60)	Gender (male:female)	Stage (I:II+ III)	Smoke (no:yes)	Tumor size (>3:≤3) (cm)	Vascular invasion (no:yes)	Lymphatic invasion (no:yes)	Differentiation (well:moderately+ poorly)
16:19	31:4	12:23	21:14	29:6	30:5	12:23	12:23

Supplementary Table 7

Primers, siRNA and mimics or inhibitors used in this study

Name	Application	Sequence
circTP63-F	qRT-PCR	GCCCTCACTCCTACAACCATT
circTP63-R	qRT-PCR	TTGTGTGCTGAGGAAGGTACT
circKRT6B-F	qRT-PCR	CTGCATTCCTGCACTGCTTTC
circKRT6B-R	qRT-PCR	CCACTTGGTGTCCAGAACCTT
circKRT5-F	qRT-PCR	AAACAGAATCCCCACCCCAA
circKRT5-R	qRT-PCR	AGCAGTGGTACGCTTGTTGA
circKRT6C-F	qRT-PCR	CTCCCTGTGATGATTTCTCTGC
circKRT6C-R	qRT-PCR	TTCTGCCTCACAGTCTTGGT
circPITX1-F	qRT-PCR	CGTCCCTGTGTATGTTGGAC
circPITX1-R	qRT-PCR	GTTACGCTCGCGCTTACG
circANKRD1-F	qRT-PCR	TGATGCGGTGAGACTGAACC
circANKRD1-R	qRT-PCR	CACGGAATTCGATCTGGGCT
TP63-F	qRT-PCR	TGGTAACAGCTCCCCACC
TP63-R	qRT-PCR	GCTCCCATGCCATCAGG
KRT6B-F	qRT-PCR	GGGAGTGGATTTGGTTT
KRT6B-R	qRT-PCR	TTGAGGGGAGTCAGGAG
KRT5-F	qRT-PCR	GAGATCGCCACTTACCG
KRT5-R	qRT-PCR	CTGCCATATCCAGAGGAA
KRT6C-F	qRT-PCR	GAGTGGATTTGGTTTCG
KRT6C-R	qRT-PCR	CTCTGGTTGACGGTGAC
PITX1-F	qRT-PCR	GTGCGGGTCTGGTTCAA
PITX1-R	qRT-PCR	CGTACACGTCCTCGTAG
ANKRD1-F	qRT-PCR	GGAACTGGTCACTGGAAAG
ANKRD1-R	qRT-PCR	CAGCAGCTTCATACTCT
U1-F	qRT-PCR	GGGAGATACCATGATCACGAAGGT
U1-R	qRT-PCR	CCACAAATTATGCAGTCGAGTTTCCC
GAPDH-F	qRT-PCR	TGCACCACCAACTGCTTAGC
GAPDH-R	qRT-PCR	GGCATGGACTGTGGTCATGAG
FOXM1-F	qRT-PCR	TTATCAGTGCTGCTAGCTGAGG
FOXM1-R	qRT-PCR	TGATGGGTCTCGCTAAGTGT
KIF18B-F	qRT-PCR	CAGTCCCCAGAGGATGAGGA
KIF18B-R	qRT-PCR	CAGTTCCCGTGCTGAGAAGT
BRCA1-F	qRT-PCR	GCTCTTCGCGTTGAAGAAGT
BRCA1-R	qRT-PCR	TGGTCACACTTTGTGGAGACA
AURKA-F	qRT-PCR	AGGAGGACCACTCTCTGTGG
AURKA-R	qRT-PCR	AGGCTCCAGAGATCCACCTT
AURKB-F	qRT-PCR	CATCCCAACATCCTGCGTCT
AURKB-R	qRT-PCR	CACCTTCTTCCCATGGCAGT

CDC25B-F	qRT-PCR	TGCTGGGATCTCATGGCCT
CDC25B-R	qRT-PCR	ATAGGGCTGGGGGAATCCAT
CENPA-F	qRT-PCR	CTTCCTCCCATCAACACAGTCG
CENPA-R	qRT-PCR	TGCTTCTGCTGCCTCTTGTAGG
CENPB-F	qRT-PCR	ATTCAGACAGTGAGGAAGAGGACG
CENPB-R	qRT-PCR	CATCAATGGGGAAGGAGGTCAG
CENPF-F	qRT-PCR	CACGAAATCCGTCCCAGTCA
CENPF-R	qRT-PCR	TTCTCACTGCCGTTGGACTC
PLK1-F	qRT-PCR	CTTCGTGTTCGTGGTGTTGG
PLK1-R	qRT-PCR	TTAGGAGTCCCACACAGGGT
CCNB1-F	qRT-PCR	TGCAGCACCTGGCTAAGAAT
CCNB1-R	qRT-PCR	TAGCATGCTTCGATGTGGCA
β-actin-F	qRT-PCR	AGTTGCGTTACACCCTTTCTTG
β-actin-R	qRT-PCR	GCTGTCACCTTCACCGTTCC
circTP63-F	plasmid construction	CGGAATTCTGAAATATGCTATCTTACAGGTGAG GGGCCGTGAGACTTAT
circTP63-R	plasmid construction	CGGGATCCTCAAGAAAAAATATATTCACTGTTG GCTCCCATGCCATC
circTP63-si-mut-F	plasmid construction	CGGAATTCTGAAATATGCTATCTTACAGTGCCG GGGAGTGGAGACTTATGAA
circTP63-si-mut-R	plasmid construction	CGGGATCCTCAAGAAAAAATATATTCACCGGTT GTTCCCATGCCATCAGG
circTP63-PGL3-F	plasmid construction	CGCCGTGTAATGTGAGGGGCCGTGAGACTTAT
circTP63-PGL3-R	plasmid construction	CGCCCCGACTGTTGGCTCCCATGCCATC
FOXM1-pGL3-F	plasmid construction	CGCCGTGTAATACTGCTGGGACCTTGTGTTC
FOXM1-pGL3-R	plasmid construction	CGCCCCGACCCACCTTCTGGCAGTCTCTG
circTP63-pGL3-mut-F	plasmid construction	CAGACCTCAATACACTGTCCACTTCATATGGTA ACAGCT
circTP63-pGL3-mut-R	plasmid construction	TGGACAGTGTATTGAGGTCTGTTTCTGAAGTAA GTGCT
FOXM1-pGL3-mut-F	plasmid construction	CATTCTCTGCCCAGCACTGTCTTACCTTCCCTGA TCTTTGCAG
FOXM1-pGL3-mut-R	plasmid construction	ACAGTGCTGGGCAGAGAATGGAAACAGGCTGG GGGGTTC
si-*circTP63*	siRNAs	GCCAACAGUGAGGGGCCGU
si-*circTP63*#2	siRNAs	CAACAGUGAGGGGCCGUGAGA
si*FOXM1*#1	siRNAs	GCAGAAACGACCGAAUCCA
si*FOXM1*#2	siRNAs	AGUGCCAACCGCUACUUGA
si*CENPA*#1	siRNAs	GGCUAAAGGAGAUCCGAAA

si*CENPA*#2	siRNAs	CACACCUCUUGAUAAGGAA
si*CENPB*#1	siRNAs	GCACGAUCCUGAAGAACAA
si*CENPB*#2	siRNAs	CCAUCACCACUUCCCUCAA
siRNA control (siNC)	siRNAs	UUCUCCGAACGUGUCACGU
has-miR-873-3p mimics	miRNA mimcs	GGAGACUGAUGAGUUCCCGGGA
mimics NC	miRNA mimcs	UCACAACCUCCUAGAAAGAGUAGA
has-miR-873-3p inhibitors	miRNA inhibitors	UCCCGGGAACUCAUCAGUCUCC
inhibitors NC	miRNA inhibitors	UCUACUCUUUCUAGGAGGUUGUGA
circTP63 Probe	Northern blots	Biotin-GGACTCTTTGATCTTCAACAGCATTTCATAAGTCTCACGGCCCCTCACTGTTGGCTCC- Biotin
Front circRNA frame	Flanking sequence	GAGTTCTAAAATTAAACTATGTGGAGTCATGTCCAACCGCACAATGCATCTTTATGTGAAACTTGCTAGAGTTTTTGTTTTCCTTCTATGTAAAAGTCCAGTTGGGAAGCTTTATTTCTGATAGATTAAATGGTATAGGTCTTTCAGTTTTCTCTTCATTTCTGACAACTGAACTGCTCTCGCCTTGAACCTGTTTTGGCACTAAAATAAAATCTGTTCAATTAACGAATTC
Back circRNA frame	Flanking sequence	GGATCCACTAATTTGGGATGATAACGCCAAAACAGGTTCAAGGCGAGAGCAGTTCAGTTGTCAGAAATGAAGAGAAAACTGAAAGACCTATACCATTTAATCTATCAGAAATAAAGCTTCCCAACTGGACTTTTACATAGAAGGAAAACAAAAACTCTAGCAAGTTTCACATAAAGATGCATTGTGCGGTTGGACATGACTCCACATAGTTTAATTTTAGAACTCAAGCCATGAACGAATAATTTTAGAG
