# Supplementary material for: circTP63 functions as a ceRNA to promote lung squamous cell carcinoma progression by upregulating FOXM1
Source: Nat Commun. 2019 Jul 19;10:3200. doi: 10.1038/s41467-019-11162-4 (PMC6642174; doi:10.1038/s41467-019-11162-4)
Supplement: Supplementary file 3 — Reporting Summary [file 41467_2019_11162_MOESM3_ESM.pdf]

nature research

Corresponding author(s): Wenxin Qin

Last updated by author(s): Jun 4, 2019

Reporting Summary

Nature Research wishes to improve the reproducibility of the work that we publish. This form provides structure for consistency and transparency in reporting. For further information on Nature Research policies, see Authors & Referees and the Editorial Policy Checklist.

Statistics

For all statistical analyses, confirm that the following items are present in the figure legend, table legend, main text, or Methods section.

n/a	Confirmed	
☐	☒	The exact sample size (*n*) for each experimental group/condition, given as a discrete number and unit of measurement
☐	☒	A statement on whether measurements were taken from distinct samples or whether the same sample was measured repeatedly
☐	☒	The statistical test(s) used AND whether they are one- or two-sided<br>*Only common tests should be described solely by name; describe more complex techniques in the Methods section.*
☒	☐	A description of all covariates tested
☒	☐	A description of any assumptions or corrections, such as tests of normality and adjustment for multiple comparisons
☐	☒	A full description of the statistical parameters including central tendency (e.g. means) or other basic estimates (e.g. regression coefficient) AND variation (e.g. standard deviation) or associated estimates of uncertainty (e.g. confidence intervals)
☐	☒	For null hypothesis testing, the test statistic (e.g. *F*, *t*, *r*) with confidence intervals, effect sizes, degrees of freedom and *P* value noted<br>*Give P values as exact values whenever suitable.*
☒	☐	For Bayesian analysis, information on the choice of priors and Markov chain Monte Carlo settings
☒	☐	For hierarchical and complex designs, identification of the appropriate level for tests and full reporting of outcomes
☒	☐	Estimates of effect sizes (e.g. Cohen's *d*, Pearson's *r*), indicating how they were calculated

Our web collection on statistics for biologists contains articles on many of the points above.

Software and code

Policy information about availability of computer code

Data collection	qRT-PCR data were collected by the ABI 7500 Fast Dx instrument's Sequence Detection Software; Western blot images were captured using Bio-Rad Image Lab software imaging software
Data analysis	Microsoft Excel and Graph Pad Prism 6 for statistical analysis and graph plotting; Modfit software was used to analyze the flow cytometry data

For manuscripts utilizing custom algorithms or software that are central to the research but not yet described in published literature, software must be made available to editors/reviewers. We strongly encourage code deposition in a community repository (e.g. GitHub). See the Nature Research guidelines for submitting code & software for further information.

Data

Policy information about availability of data

All manuscripts must include a data availability statement. This statement should provide the following information, where applicable:

- Accession codes, unique identifiers, or web links for publicly available datasets
- A list of figures that have associated raw data
- A description of any restrictions on data availability

This microarray data are deposited in the NCBI Gene Expression Omnibus (GEO) datasets under the accession number GSE126533. The authors declare that all the data supporting the findings in this study are available in this study and its Supplementary Information.The source data underlying Figs 4e-f, 5g and 6a-b and Supplementary Figs 5c and 6c are provided as a Source Data file.

Field-specific reporting

Please select the one below that is the best fit for your research. If you are not sure, read the appropriate sections before making your selection.

☒ Life sciences ☐ Behavioural & social sciences ☐ Ecological, evolutionary & environmental sciences

For a reference copy of the document with all sections, see nature.com/documents/nr-reporting-summary-flat.pdf

Life sciences study design

All studies must disclose on these points even when the disclosure is negative.

Sample size	Sample-size calculations were not required for all experiments of this study. 5 paired samples of LUSC patients were used for Microarray analysis; 35 paired samples of LUSC patients were used for qRT-PCR validation.
Data exclusions	No data have been excluded from the analysis.
Replication	All in vitro experiments were repeated at least three independent times. For xenograft experiments, 6 or more biologically independent tumors were used.
Randomization	For in vitro study, cells were based on gain or loss of function experiments with appropriate controls. For in vivo study, animal injected with H2170 and H1703 cells were chosen randomly. Animals with xenograft tumours treated with cholesterol-conjugated si-NC or si-circTP63 were divided randomly.
Blinding	All experiments were performed in non-blinded manner

Reporting for specific materials, systems and methods

We require information from authors about some types of materials, experimental systems and methods used in many studies. Here, indicate whether each material, system or method listed is relevant to your study. If you are not sure if a list item applies to your research, read the appropriate section before selecting a response.

Materials & experimental systems

n/a	Involved in the study	
☐	☒	Antibodies
☐	☒	Eukaryotic cell lines
☒	☐	Palaeontology
☐	☒	Animals and other organisms
☒	☐	Human research participants
☐	☒	Clinical data

Methods

n/a	Involved in the study	
☒	☐	ChIP-seq
☐	☒	Flow cytometry
☒	☐	MRI-based neuroimaging

Antibodies

Antibodies used	FOXM1 (1:3000 dilution; proteintech), CENPA (1:1000 dilution; Abcam), CENPB(1:1000 dilution; Abcam), CCNB1 (1:1000 dilution, Cell Signaling Technology),β-actin (1:10000 dilution; Sigma)
Validation	All antibodies were validated by the supplier for human samples, and were checked in the lab by Western blotting on cell lysate and by comparing to the manufacturer's results.

Eukaryotic cell lines

Policy information about cell lines

Cell line source(s)	Human cell lines(NCI-H2170, NCI-H1703, NCI-H226, NCI-H520,<br>14 SW900, SK-MES-1, BEAS-2B and HFL-1) used in this study were described in Methods section. They were acquired from ATCC.
Authentication	Cell lines were obtained from original sources and were not further authenticated.
Mycoplasma contamination	All cell lines used in the study were tested negative for mycoplasma contamination
Commonly misidentified lines<br>(See ICLAC register)	does not apply

Animals and other organisms

Policy information about studies involving animals; ARRIVE guidelines recommended for reporting animal research

Laboratory animals	Six to eight-week-old male BALB/c nude mice
Wild animals	does not apply
Field-collected samples	does not apply
Ethics oversight	Mouse experiments were approved by the Shanghai Medical Experimental Animal Care Commission.

Note that full information on the approval of the study protocol must also be provided in the manuscript.

Clinical data

Policy information about clinical studies

All manuscripts should comply with the ICMJE guidelines for publication of clinical research and a completed CONSORT checklist must be included with all submissions.

Clinical trial registration	does not apply
Study protocol	does not apply
Data collection	does not apply
Outcomes	does not apply

Flow Cytometry

Plots

Confirm that:

☐ The axis labels state the marker and fluorochrome used (e.g. CD4-FITC).

☒ The axis scales are clearly visible. Include numbers along axes only for bottom left plot of group (a 'group' is an analysis of identical markers).

☒ All plots are contour plots with outliers or pseudocolor plots.

☒ A numerical value for number of cells or percentage (with statistics) is provided.

Methodology

Sample preparation	This information is included in the methods section page.
Instrument	flow cytometry (Beckman FC500, Los Angeles, CA, USA)
Software	Modfit software
Cell population abundance	> 10,000 cells per experiment were analyzed
Gating strategy	FSC-A/SSC-A gates of the starting cell population were used to dicriminate between viable cells and cells deribris. Singlet and doublet cells were discriminated using FSC-A/FSC-W gating

☒ Tick this box to confirm that a figure exemplifying the gating strategy is provided in the Supplementary Information.
